# Early diastolic functional abnormalities in normotensive offspring of Nigerian hypertensives

**DOI:** 10.5830/CVJA-2011-030

**Published:** 2012-06

**Authors:** AM Adeoye, AA Adebiyi, OO Oladapo, OS Ogah, A Aje, DB Ojji, AK Adebayo, KC Ochulor, EO Enakpene, AO Falase

**Affiliations:** Department of Medicine, University College Hospital, Ibadan, Nigeria; Department of Medicine, College of Medicine, University of Ibadan, Ibadan, Nigeria; Department of Medicine, University College Hospital, Ibadan, Nigeria; Department of Medicine, College of Medicine, University of Ibadan, Ibadan, Nigeria; Department of Medicine, University College Hospital, Ibadan, Nigeria; Department of Medicine, College of Medicine, University of Ibadan, Ibadan, Nigeria; Department of Medicine, University College Hospital, Ibadan, Nigeria; Department of Medicine, University College Hospital, Ibadan, Nigeria; Department of Medicine, University College Hospital, Ibadan, Nigeria; Department of Medicine, University College Hospital, Ibadan, Nigeria; Department of Medicine, University College Hospital, Ibadan, Nigeria; Department of Medicine, University College Hospital, Ibadan, Nigeria; Department of Medicine, University College Hospital, Ibadan, Nigeria; Department of Medicine, College of Medicine, University of Ibadan, Ibadan, Nigeria

**Keywords:** diastolic function, offspring, hypertension, Nigerian

## Abstract

**Background:**

Some studies have suggested that diastolic dysfunction precedes the clinical manifestation of hypertension. Whether changes in cardiac structure and function predate the clinical manifestation of hypertension later in life is now being investigated. The aim of this study was to assess the differences in cardiac structure and function between the offspring of hypertensive and normotensive parents.

**Methods:**

Eighty normotensive offspring of hypertensive parents (OHyp) (41 females and 39 males) and 62 normotensive offspring of normotensive parents (ONorm) (31 males and 31 females) were recruited for echocardiography.

**Results:**

The mean age was 25.0 (5.31) and 24.3 (3.60) years in the OHyp and ONorm participants, respectively (*p* = 0.369). Other baseline parameters were comparable between the two groups. Septal wall thickness in systole was higher in the OHyp than the ONorm subjects [1.3 (0.35) vs 1.1 (0.25), *p* = 0.0173]. Indexed left ventricular mass [28.1 (7.33) vs 27.5 (7.23), *p* = 0.631] and relative wall thickness [(0.3 (0.10) vs 0.3 (0.90), *p* = 0.280] were similar in the two groups. The offspring of hypertensives had lower deceleration time [149.9 (38.89) vs 169.0 (50.08) ms, *p* = 0.012], prolonged duration of pulmonary A reverse flow [113.5 (70.69) vs 81.7 (38.31) ms, *p* = 0.024], increased myocardial isovolumic relaxation time [173.4 (47.98) vs 156.1 (46.74) ms, *p* = 0.033] and a lower myocardial Em [0.2 (0.05) vs 0.3 (1.38), *p* = 0.037] and myocardial Em/Am ratio [1.6 (0.01) vs 2.1 (0.01), *p* = 0.019] than the offspring of normotensives.

**Conclusion:**

This study showed that offspring of OHyp subjects had early diastolic functional abnormalities when compared with offspring of ONorm participants. Longitudinal studies are needed to determine the implications of this finding in this African population.

## Abstract

Worldwide, hypertension is an independent risk factor for cardiovascular morbidity and mortality.^1^ Hypertension is the most common non-communicable disease in Nigeria, a typical example of a developing country. Despite innovations in the drug-related management of hypertension, control remains poor.^2^ Less than a third of individuals with a usual blood pressure exceeding 140/90 mmHg are adequately treated.^3^ For this reason, global approaches now tend to focus on lifestyle changes and studies directed at the aetiopathogenesis of hypertension. The aim is to find markers for the early detection of hypertension so as to initiate preventive as well as control measures as widely as possible.

Studies on offspring of hypertensive patients have shown the significant roles of heredity, salt intake, increased peripheral vascular resistance, insulin resistance and increased left ventricular (LV) mass in the pathogenesis of hypertension.^4^-^6^ Increased LV mass and diastolic dysfunction can be either a consequence of hypertension or precede the clinical manifestation of hypertension.^7^-^10^ Diastolic dysfunction has been demonstrated in borderline hypertensive and normotensive offspring of hypertensive patients in the absence of increased LV mass.^10^-^16^

Since most of these studies have been carried out in Europe and America, little is known about LV filling patterns in the offspring of hypertensive Nigerians, in a country with increasing prevalence of hypertension, as are most other African countries. This study therefore aimed to determine LV diastolic filling patterns in normotensive offspring of hypertensive Nigerians in comparison with normotensive control subjects without a family history of hypertension.

## Methods

The study was carried out at the cardiology unit of the Department of Medicine of the University College Hospital, Ibadan, Nigeria. Eighty normotensive offspring aged 18 to 40 years with hypertensive parents attending the cardiac clinic of the Hospital were recruited over a six-month period. Subjects were offspring of consecutive hypertensive parents seen at the medical outpatient department.

Comparable control subjects were recruited among the children of normotensive hospital staff and relatives of patients on treatment for conditions other than hypertension or other chronic medical conditions. Ethical approval was obtained from the institutional ethics review board and informed consent was obtained from each participant.

Blood pressure was measured with a mercury sphygmomanometer (Accosson) according to standard guidelines.^17^ Systolic and diastolic blood pressure was measured at Korotokoff sound phases 1 and V, respectively. Subjects with blood pressure higher than 140/90 mm/Hg and body mass index (BMI) above 25 kg/m^2^ were excluded from the study. Other exclusion criteria were existing heart disease and diabetes mellitus.

Echocardiographic examination was performed with the subjects in partial left lateral decubitus position using an Aloka SSD1700 machine (Aloka Co. Ltd, Tokyo, Japan) with a 3.5-MHz transducer. Two-dimensional guided M-mode measurements were obtained as recommended by the American Society of Echocardiography.^18^ Left ventricular (LV), septal, posterior wall thickness and cavity dimensions were measured using leading-edge methodology at both end-diastole and end-systole. Left ventricular mass (LVM) was calculated using the formula of Devereux and Reichek.^19^ This has been shown to yield LVM closely related to autopsy measurements (*r* = 0.90),^20^ and had good inter-observer reproducibility (*p* = 0.93) in one study.^21^

LVM was indexed by the allometric power of height (LVM/Ht^2.7^.^22^ Left ventricular hypertrophy was considered present if the left ventricular mass index (LVMI) was ≥ 46 g/m^2.7^. Relative wall thickness (RWT) was calculated as 2RWTd/LVIDd (left ventricular internal diameter).^7^ Increased wall thickness was present when RWT > 0.45.^23^ Ejection fraction was calculated using the formula of Teichholz.^24^

Doppler echocardiography was used for transmitral flow velocities, obtained with the Doppler sample volume placed just beyond the tip of the mitral valve leaflets. The parameters measured were early diastolic peak flow velocity (E), early diastolic flow time (EDFT), late diastolic peak flow velocity (A), late diastolic flow time (ADFT), the deceleration time of early mitral velocity, and the ratio of E to A (E/A).

Isovolumic relaxation time (IVRT) was measured with the pulse-wave Doppler beam intersecting the LV outflow and inflow tracts. EDFT was measured from the onset of diastolic flow to the intersection of a line extrapolated to the baseline, and ADFT was measured from the onset of late diastolic flow to the end of diastolic flow.^25^

Pulmonary venous flow recordings were obtained from a four-chamber view directed at the right upper pulmonary vein. The sample volume was placed 1–2 cm into the pulmonary vein and the following measurement were recorded: peak S-wave velocity (peak systolic pulmonary venous inflow velocity during ventricular systole), peak D-wave velocity (peak diastolic pulmonary venous inflow velocity during early phase of diastole), peak AR-wave velocity (peak reversed systolic wave during atrial contraction), and duration of the reverse atrial contraction-induced diastolic flow.^26^

Myocardial Doppler velocities were measured in the apical four-chamber view with the Doppler beam well aligned to the septum and the pulsed Doppler sample volume placed 1 cm apically from the mitral annulus in the interventricular septal myocardium. The following measurements were made: myocardial isovolumic contraction time, myocardial peak systolic velocity (Sm), myocardial contraction time, myocardial isovolumetric relaxation time, myocardial early diastolic relaxation velocity (Em), and myocardial late relaxation velocity (Am). Other measurements were the duration of the diastolic period and the durations of Em and Am. Measurements from three cardiac cycles were taken and averaged.

The calculation of the sample size for this study was based on a difference of 0.2 in the mitral E/A ratio between the controls and the subjects, with a 90% power to detect the difference at a significance level of 0.05 in a two-tailed test. The estimated sample size was 63 subjects per group. [This was based on data from a previous study where LVM (± SD) in hypertensive and normotensive offspring was 125 (29) and 109 (25) g, respectively.]^27^

## Statistical analysis

All data generated were entered into a standard proforma. SPSS software, version 10.0 (SPSS Inc., Chicago, Illinois) was used for statistical analysis. Continuous variables were expressed as mean (standard deviation). Differences in continuous variables between the groups were assessed with a *t*-test for independent groups. Where data were not normal, the Mann-Whitney test was used to compare the two groups. Data were adjusted for covariates using analysis of covariance. A two-tailed *p*-value < 0.05 was considered significant.

## Results

Table 1 shows the baseline characteristics of the participants (subjects and controls). Systolic and diastolic pressure, mean arterial pressure, age, height and body mass index were comparable in the two groups.

**Table 1.** Baseline Characteristics Of The Subjects

*Characteristics*	*Offspring of hypertensive parents (n = 80)*	*Offspring of normotensive parents (n = 62)*	p*-value*
Age (years)	25.0 (5.31)	24.3 (3.60)	0.369
Weight (kg)	64.2 (10.87)	63.2 (9.93)	0.565
Height (cm)	1.7 (0.10)	1.7 (0.10)	0.856
Body mass index (kg/m^2^)	22.9 (3.07)	22.6 (2.51)	0.499
Body surface area (m^2^)	1.7 (0.18)	1.7 (0.17)	0.659
Waist circumference (cm)	79.8 (9.12)	77.6 (7.21)	0.177
Hip circumference (cm)	97.2 (8.78)	94.6 (8.85)	0.121
Waist–hip ratio	0.8 (0.05)	0.8 (0.07)	0.838
Systolic blood pressure (mmHg)	115.0 (12.88)	111.7 (10.08)	0.122
Diastolic blood pressure (mmHg)	72.8 (8.57)	70.5 (8.76)	0.131
Pulse pressure (mmHg)	42.2 (11.35)	41.2 (10.19)	0.789
Mean arterial pressure (mmHg)	86.9 (8.70)	84.3 (7.87)	0.067
Heart rate	76.6 (12.90)	76.8 (12.30)	0.909

The cardiac structure and systolic functional data are listed in Table 2. Apart from the left atrial diameter and septal wall thickness at systole that was greater in the offspring of hypertensive subjects, relative LV wall thickness, LVM, LVMI, aortic root diameter, left atrial diameter, LV internal diameters and LV ejection fraction were similar in both groups.

**Table 2.** Measured Echocardiographic Characteristics

*Variable (cm)*	*Offspring of hypertensive parents (n = 80)*	*Offspring of normotensive parents (n = 62)*	p*-value*	*Adjusted p-value*
Aortic root diameter	2.5 (0.29)	2.5 (0.27)	0.880	0.655
Left atrial diameter	3.1 (0.43)	3.0 (0.45)	0.197	0.042*
IVSTd	0.9 (0.19)	0.9 (0.19)	0.642	0.989
IVSTs	1.3 (0.35)	1.1 (0.25)	0.017*	0.038*
PWTd	0.7 (0.18)	0.7 (0.17)	0.248	0.658
PWTs	1.1 (0.25)	1.2 (0.24)	0.100	0.108
LVIDd	4.4 (0.47)	4.3 (0.47)	0.811	0.151
LVIDs	2.8 (0.46)	2.8 (0.38)	0.919	0.844
Fractional shortening (%)	35.0 (7.60)	35.0 (5.90)	0.585	0.180
Ejection fraction (%)	64.0 (9.80)	63.0 (7.80)	0.734	0.220
Ejection time (ms)	357.0 (46.20)	375.0 (45.80)	0.042*	0.043*
Absolute LVM (g)	112.6 (31.60)	110.7 (33.90)	0.736	0.212
LVM/Ht (g/m^2.7^)	28.1 (7.33)	27.5(7.23)	0.631	0.129
RWT	0.3 (0.09)	0.3 (0.08)	0.280	0.277

LVM = left ventricular mass, BSA = body surface area, RWT = relative wall thickness, Ht = height. *Statistically significant. Data adjusted for age, systolic blood pressure, diastolic blood pressure, body surface area and body mass index.

Table 3 shows the echocardiographic diastolic functional indices in both groups. The duration of the E wave and pulmonary A reversal flow was significantly higher in the offspring of hypertensive subjects than in the controls. The deceleration time of the E wave (DT) was lower in offspring of hypertensive subjects.

**Table 3.** Echocardiographic Doppler Indices Of LV Diastolic Function In Subjects And Controls

*Variable*	*Offspring of hypertensive parents (n = 80)*	*Offspring of normotensive parents (n = 62)*	p*-value*	*Adjusted p-value*
Transmitral E wave (m/s)	0.7 (0.17)	0.7 (0.13)	0.938	0.809
Duration of E wave (ms)	207.3 (45.35)	228.7 (50.40)	0.009*	0.020
Deceleration time of E wave (ms)	149.9 (38.89)	169.0 (50.08)	0.012*	0.025
A-wave velocity (m/s)	0.4 (0.13)	0.4 (0.09)	0.123	0.100
Isovolumetric relaxation time (IVRT)	121.0 (30.82)	113.8 (23.14)	0.378	0.148
E/A ratio	1.9 (0.51)	2.1 (0.61)	0.062	0.122
Pulmonary S wave (m/s)	0.5 (0.18)	0.4 (0.17)	0.848	0.937
Pulmonary D wave (m/s)	0.3 (0.10)	0.3 (0.10)	0.740	0.655
S/D ratio	1.5 (.0.44)	1.5 (0.54)	0.820	0.965
Pulmonary A wave (m/s)	0.2 (0.09)	0.2 (0.06)	0.652	0.545
Duration A reverse wave (ms)	113.5 (70.69)	81.7 (49.31)	0.024*	0.067

*Statistically significant. Data adjusted for age, systolic blood pressure, diastolic blood pressure, body surface area and body mass index.

Table 4 shows the measured tissue Doppler parameters. Offspring of hypertensive subjects had a higher myocardial isovolumetric relaxation time as well as a lower myocardial E velocity and E/A ratio. The diastolic period was also longer in the OHyp subjects compared to controls.

**Table 4.** Tissue Doppler Indexes At The Medial (Septal) Mitral Valve Annulus

*Variable (medial annulus)*	*Offspring of hypertensive parents (n = 80)*	*Offspring of normotensive parents (n = 62)*	p*-value*	*Adjusted p-value*
Medial Sm (m/s)	0.1 (0.02)	0.1 (0.04)	0.970	0.940
Medial Em (m/s)	0.2 (0.05)	0.3 (1.38)	0.037*	0.348
Medial Am (m/sec)	0.1 (0.03)	0.1 (0.03)	0.844	0.882
Medial Em/Am	1.6 (0.01)	2.1 (0.01)	0.019*	0.028
Duration of Sm (ms)	210.9 (40.58)	201.6 (42.06)	0.186	0.187
Duration of Em (ms)	133.7 (28.87)	128.0 (26.05)	0.227	0.156
Duration of Am (ms)	85.4 (18.40)	85.1 (27.06)	0.954	0.860
Duration of diastolic period (ms)	420.6 (136.89)	401.8 (139.32)	0.424	0.390
Isovolumetric relaxation time (IVRT) (ms)	156.1 (46.74)	173.4 (47.98)	0.033*	0.031
Isovolumetric contraction time (IVCT) (ms)	115.9 (44.09)	113.5 (38.01)	0.731	0.740

Among the male gender, statistical differences were found in only some echocardiographic parameters. Hypertensive vs normotensive offspring: left atrial diameter [3.20 (0.42) vs 2.93 (0.44) cm, *p* = 0.012]; posterior wall thickness in systole [1.13 (0.26) vs 1.29 (0.21) cm, *p* = 0.008]; and deceleration time of the E wave [180.8 (60.2) vs 152.7 (44.6) ms, *p* = 0.034].

On the other hand, among the females, statistical differences were found in some of the physical and echocardiographic parameters. Hypertensive vs normotensive offspring: height [1.64 (0.08) vs 1.60 (0.05) m, *p* = 0.032]; body surface area [1.66 (0.17) vs 1.59 (0.13) m^2^, *p* = 0.040]; diastolic blood pressure [71.1 (8.3) vs 671.(7.5) mmHg, *p* = 0.040]; mean arterial blood pressure [84.5 (9.0) vs 80.6 (6.5) mmHg, *p* = 0.040]; duration of the medial annulus IVRT [178.0 (55.4) vs 151.2 (54.5) ms, *p* = 0.045].

## Discussion

This study shows that normotensive offspring of hypertensive Nigerians had abnormal diastolic functional parameters compared with normotensive control subjects without a family history of hypertension. The changes in left ventricular filling dynamics and an increased left ventricular mass occurred early in the development of systemic hypertension. Increased left ventricular mass and diastolic dysfunction have been demonstrated in the early stages of hypertension, in the stage of prehypertension, and in hypertensive subjects without left ventricular hypertrophy.

In this study, the offspring of hypertensive subjects showed features of early diastolic dysfunction even in the absence of increased left ventricular mass. This was demonstrated by significantly higher deceleration time of E, as well as a prolonged diastolic period, assessed by tissue Doppler. Impaired relaxation is conventionally associated with increased A velocity, and reduced E/A ratio. Our finding was similar to the work of Graettinger *et al*.^16^ who also demonstrated that the flow time and the time integral of the A wave were higher in the offspring of hypertensives, implying a shift towards late diastolic filing.

Pulmonary vein flow parameters are useful non-invasive methods of assessment of LV diastolic function. The present study showed prolonged duration of the A reverse flow (Ar) in the offspring of hypertensives. Prolonged Ar duration is associated with impaired relaxation, as well as reduced preload.^28^ There were no demonstrable changes in the loading condition, hence the prolonged duration of Ar can only be explained by impaired relaxation. Transmitral inflow measurements may be affected by age, BMI, LV mass, and heart rate but all these variables were comparable in the two groups,^29^-^31^ and were corrected for.

Tissue Doppler imaging is a non-invasive and easily reproducible method of assessment of left ventricular function. The parameters are less influenced by preload changes compared with transmitral and pulmonary vein flow measurements and also believed to be more sensitive than the convectional Doppler methods. Our septal myocardial tissue Doppler measurements showed reduced Em and Em/Am ratio and increased IVRT in the offspring of hypertensives. These findings are indicative of diastolic dysfunction and were similar to those of Aeschbacher *et al*.^32^ in their prospective study of the offspring of hypertensive subjects, particularly offspring who became hypertensive after five years of follow up.

In an earlier study by the same group,^33^ on offspring of hypertensives, there was no evidence of diastolic dysfunction using conventional Doppler methods. At follow up, pulmonary vein flow and myocardial tissue imaging were added and despite comparable blood pressure and left ventricular masses, the diastolic dysfunction became more evident. Our study confirms that if sensitive methods of assessing diastolic function are employed, deterioration in diastolic function can be demonstrated in normotensive subjects with a parental history of hypertension. This further confirms the hypothesis that abnormalities of cardiac function may predate clinical hypertension in genetically predisposed individuals.

Studies that did not demonstrate differences between diastolic function in the offspring of hypertensives and normotensives used only mitral inflow parameters, which are inadequate in assessing diastolic function, as the parameters are easily affected by loading conditions.^13^,^14^ Our study had the advantage of assessing diastolic function in offspring of hypertensives using conventional Doppler parameters as well as myocardial tissue Doppler imaging.

Our data suggest that diastolic dysfunction was present in offspring of hypertensive subjects despite the fact that they had similar baseline characteristics and left ventricular dimensions to the control group. Although the effect of the insignificantly higher blood pressure and LVM on the findings of diastolic function cannot be totally ruled out, our findings are similar to those of Aeschbacher *et al*,^33^ who also corrected for these variables in the analysis.

Gender differences noted in the analysis are not surprising. Other workers have reported gender differences in LV parameters, as well as parameters of LV diastolic function.^33^-^36^ In a study of 121 normotensive youths, Kapuku *et al*.^36^ noted that females had a higher relative wall thickness, lower E/A ratio and shorter IVRT than males.

Before changes in LV mass or hypertension become apparent, various changes occur at the cellular level that cause alterations in the myocardial architecture and collagen structure. Metabolic and neuro-endocrine changes also occur. Some studies have demonstrated acceleration of these factors in the offspring of hypertensives.

The study had some limitations. We did not obtain information on and correct for factors such as socio-economic status, level of physical activity, diet or biochemical parameters, and this may have affected the results.

## Conclusion

Diastolic dysfunction has been shown to affect the quality of life of patients with advanced cardiac disease. Assessment of diastolic function is of paramount importance in the management of this disease. Early identification of subjects with or at risk for hypertension and diastolic dysfunction may help to stratify risk, guide therapy and prevent target-organ damage in these patients.

The consistent independent effects of increased body weight and particularly male gender^37^,^38^ on the development of increased left ventricular mass reaffirms the need for early intervention with the institution of lifestyle modification in these subjects. Lifestyle modifications have been shown to reduce blood pressure in hypertensive patients. These interventions may prevent or delay the development of hypertension in normotensive subjects in the long term. Longitudinal studies are needed to determine the prognosis of these changes in normotensive offspring of hypertensive parents.
